# Imatinib Mesylate-Loaded Rosin/Cinnamon Oil-Based In Situ Forming Gel against Colorectal Cancer Cells

**DOI:** 10.3390/gels8090526

**Published:** 2022-08-23

**Authors:** Ei Mon Khaing, Torsak Intaraphairot, Jongjan Mahadlek, Siriporn Okonogi, Wiwat Pichayakorn, Thawatchai Phaechamud

**Affiliations:** 1 Programme of Pharmaceutical Engineering, Faculty of Pharmacy, Silpakorn University, Nakhon Pathom 73000, Thailand; 2 Department of Biopharmacy, Faculty of Pharmacy, Silpakorn University, Nakhon Pathom 73000, Thailand; 3 Natural Bioactive and Material for Health Promotion and Drug Delivery System Group (NBM), Faculty of Pharmacy, Silpakorn University, Nakhon Pathom 73000, Thailand; 4 Pharmaceutical Intellectual Center “Prachote Plengwittaya”, Faculty of Pharmacy, Silpakorn University, Nakhon Pathom 73000, Thailand; 5 Research Center of Pharmaceutical Nanotechnology, Faculty of Pharmacy, Chiang Mai University, Chiang Mai 50200, Thailand; 6 Department of Pharmaceutical Sciences, Faculty of Pharmacy, Chiang Mai University, Chiang Mai 50200, Thailand; 7 Department of Pharmaceutical Technology, Faculty of Pharmaceutical Sciences, Prince of Songkla University, Songkhla 90110, Thailand; 8 Department of Pharmaceutical Technology, Faculty of Pharmacy, Silpakorn University, Nakhon Pathom 73000, Thailand

**Keywords:** rosin, in situ forming gel, cinnamon oil, imatinib mesylate, colorectal cancer

## Abstract

Localized delivery systems have been typically designed to enhance drug concentration at a target site and minimize systemic drug toxicity. A rosin/cinnamon oil (CO) in situ forming gel (ISG) was developed for the sustainable delivery of imatinib mesylate (IM) against colorectal cancer cells. CO has been claimed to express a potent anticancer effect against various cancer cells, as well as a synergistic effect with IM on colorectal cancer cells; however, poor aqueous solubility limits its application. The effect of rosin with the adding CO was assessed on physicochemical properties and in vitro drug release from developed IM-loaded rosin/CO-based ISG. Moreover, in vitro cytotoxicity tests were conducted against two colorectal cancer cells. All formulations exhibited Newtonian flow behavior with viscosity less than 266.9 cP with easier injectability. The adding of CO decreased the hardness and increased the adhesive force of the obtained rosin gel. The gel formation increased over time under microscopic observation. CO-added ISG had a particle-like gel appearance, and it promoted a higher release of IM over a period of 28 days. All tested ISG formulations revealed cytotoxicity against HCT-116 and HT-29 cell lines at different incubation times. Thus, CO-loaded rosin-based ISG can act as a potentially sustainable IM delivery system for chemotherapy against colorectal cancer cells.

## 1. Introduction

Colorectal cancer, also referred to as bowel cancer, is one of the most common cancers [1,2]. Globally, it ranks as the third most common cancer, with 1.4 million new cases and causing over 700,000 deaths per annum [2,3,4]. Apparently, its incidence peak at 60–70 years of age affects males slightly more than females [4]. Its pathogenesis includes genetic and epigenetic abnormalities that often grow as polypoid masses (in the cecum and ascending colon) or as annular lesions that cause distal colon obstruction [5,6,7]. Polyps can be further classified histologically into three main categories, of which neoplastic polyps (adenomas) are of the utmost importance to this cancer [8]. Nowadays, the cost of colorectal cancer treatment places a significant economic burden on populations and healthcare systems, such as surgical costs ranging from USD 1149 to 34,606, chemotherapy ranging from USD 1883 to 18,021, and radiotherapy ranging from USD 2037 to 5347 in 2018. Therefore, these costs actually impact healthcare budgets and guide policymakers in making informed decisions [9]. The current treatment for colorectal cancer depends on the type and stage of disease and includes surgery, chemotherapy, immunotherapy, and radiotherapy. Chemotherapy has been shown to be a highly effective anticancer therapy among the current treatment options when used alone or in combination with other drugs [10,11]. Imatinib mesylate (IM) is a chemotherapeutic agent in which tyrosine kinase inhibitor inhibiting platelet-derived growth factor receptor (PDGFR) acts as the mechanism of action. This drug is employed to treat gastrointestinal stromal tumors and chronic myeloid leukemia [12] and it presents the potent anticancer activities against colorectal cancer cells [13,14]. Nonetheless, the conventional chemotherapeutic agents induce different serious side effects. In addition, they are expensive with low bioavailability and chemoresistant property. Therefore, several studies have been developed in regard to a polymeric drug delivery system to overcome the shortcomings of traditional formulations [10,15].

Cinnamon oil (CO) from cinnamon bark is a volatile oil comprising cinnamaldehyde [16]. Several researchers have identified various biological activities of cinnamon extract and CO, including anticancer, antifungal, antibacterial and antioxidant ones [17,18,19,20]. CO also exhibits potent cytotoxic activity against human colorectal cancer cells. Previous studied found that the combination of IM and CO exhibited a synergistic effect on colorectal cancer cell lines and also improved the efficacy of IM [21]. However, the applications of some oils in the dosage forms are limited due to their low solubility [22]. Therefore, it is important to utilize the most effective strategies for applying CO to colorectal cancer cells. The in situ forming gel (ISG) system is a localized depot drug delivery system in which various polymers or biomaterials have been employed as the matrix forming agents, whereas peppermint oil and clove oil have been included as being able to modify antibiotic drug release [23,24,25]. Nevertheless, the physicochemical properties of the CO-loaded rosin-based ISG system for delivering chemotherapeutic agents such as IM have not been investigated previously.

Solvent exchange-induced in situ forming gel (ISG) is a localized injectable drug delivery system that has the potential to deliver the drug because of its ability to sustain drug release and make the drug level sufficiently for achieving the pharmacological effect at the target site [23,26]. ISG is an injectable polymeric drug delivery system whose active ingredients dissolve in biocompatible solvents. It transforms from a solution state into a gel or a matrix-like state after being injected into an aqueous environment via a solvent exchange mechanism where the entrapped drugs are liberated from the precipitated gel over time [26]. Owing to its solution state initially, ISG can be administered via injection easily with less pain [27]. These systems can deliver the drug locally to improve its concentration around the injection site and reduce systemic toxicity [28,29,30]. Recent research indicated that paclitaxel-loaded ISG systems controlled the drug release rate and showed a cytotoxic effect against HepG2 cells [31]. Additionally, the local injection of doxorubicin-loaded zein-based in situ gel enhanced its anti-cancer efficacy and reduced the side effects [32]. It can be transformed into a semi-solid after intra-tumoral injection to BALB/c nude tumor-bearing mice. The efficient accumulation of DOX in the tumor with a lower drug concentration in blood and normal organs was obtained resulting in the effective inhibition of tumor growth and fewer off-target side effects [33]. The intra-tumoral injection of 5-Fu-loaded MPEG-b-(PCL-ran-PLLA) diblock copolymer solution rapidly gelled in vivo and resulted in the significant suppression of tumor growth in the abdomen of inoculated melanoma C57BL/6 mice [34]. The ISG system can be administered to deliver the chemotherapeutic drug to the colorectal cancer via the intra-tumoral injection, rectal route or intraperitoneally [10,35,36]. Rosin has attracted attention for use as a carrier in the ISG system because of its biocompatible, biodegradable and cost-effective properties [25]. Rosin also has film-forming properties as well as anticancer and antimicrobial activities [37,38,39,40]. It has been used as a matrix-forming agent in various drug delivery systems, such as microparticles, and nanoparticles for controlled drug delivery [41,42,43,44]. The rosin-based ISG system with adding lime peel oil could modulate rosin gel formation, drug release and inhibition zone of antimicrobial activities [45]. Therefore, the role of CO addition on the rosin-based ISG systems on their physicochemical properties to deliver IM and to improve the inhibition of cancer should be investigated.

In this research, IM-loaded rosin/CO-based ISG systems were designed, and their physicochemical properties were assessed, including rheological properties, mechanical properties, microscopic observation of gel formation, and drug release. In vitro cytotoxicity against HCT-116 and HT-29 colorectal cancer cells was also investigated.

## 2. Results and Discussion

### 2.1. Rheological Characterization

**Figure 1:**
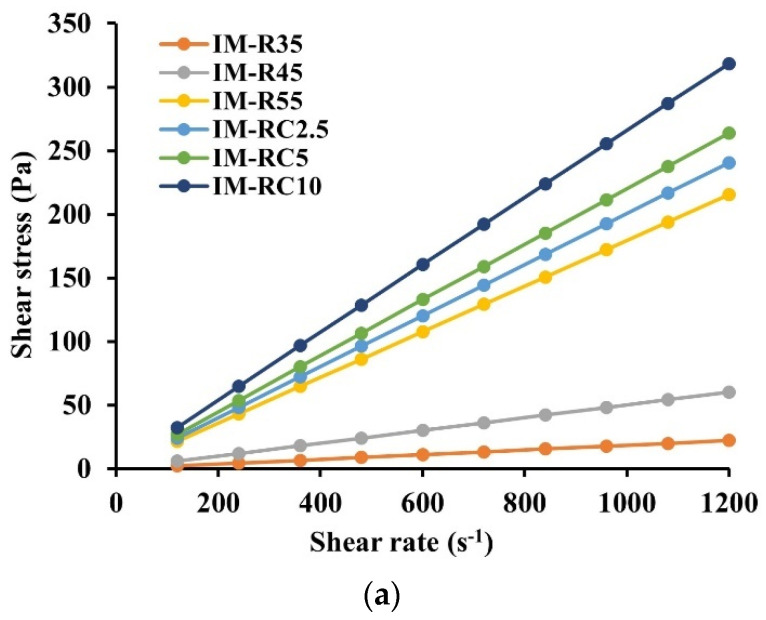

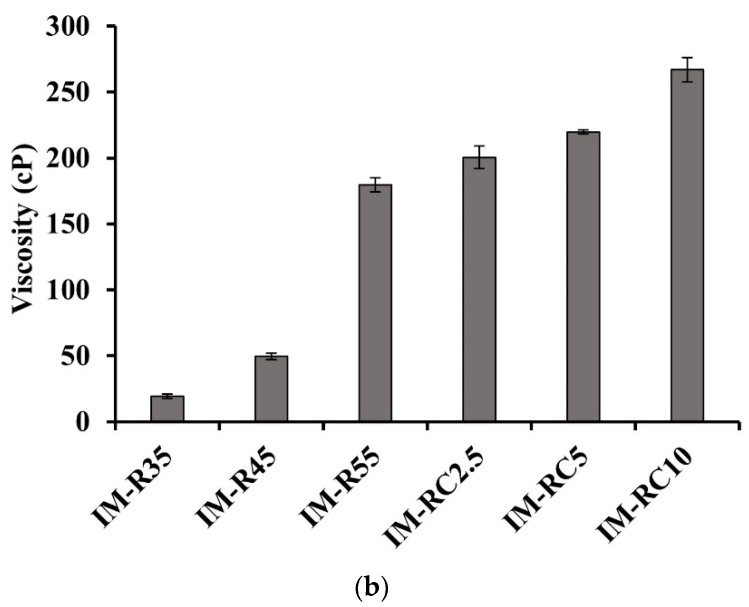
Relationship between the shear stress and shear rates (**a**) and viscosity (**b**) of IM-loaded rosin/CO-based ISG formulations at 25 °C. The data are represented in triplicates.

From the rheological examination, various ISG formulations show a linear relationship between shear stress and shear rate (Figure 1a); therefore, all prepared formulations exhibited a Newtonian flow behavior (Figure 1a). These results agree with previous research on the Newtonian behavior of borneol-based ISG [46]. The oil addition did not influence the flow of ISG systems; though, the stress on formulations increased when the oil amount was increased [23]. These results confirm the Newtonian flow behavior of oil-adding ISG system as previously reported [15,16].

Figure 1b shows the effect of rosin and CO concentrations of ISG formulations on viscosity. Increasing the rosin concentration enhanced the viscosity of the ISG formulations. The viscosities of the ISG formulations for IMR35, IMR45 and IMR55 were 19, 49 and 179 cP, respectively. The viscosity of the ISG systems notably increased as the CO concentration increased from 2.5 to 10% (IM-RC2.5, IM-RC5 and IM-RC10). It was found that the viscosity values of the CO-added ISG formulations significantly increased (*p*  <  0.05) compared to IMR55. These prepared ISG systems showed a viscosity value of less than that of 5% doxycycline hyclate-loaded rosin/lime-peel-oil-based ISG system, whose values were in the range of 624.51–684.15 cP [45]. The hydrophobic nature of CO promoted a more viscous character, which was consistent with previous research [25]. Additionally, the solvent exchange-induced ISG system presented a lower viscosity than the hydrogel-based injectable ISG system [47]. In this study, the viscosity of all prepared ISG systems was appropriate for injection because of a viscosity threshold of less than 300 mPas [48]. Typically, the lower viscosity of polymeric solution is attained when a solvent with a high potential to dissolve that polymer is used. In addition, the viscosity value was lower than other injectable thermo-responsive in situ forming gel systems, such as carbopol–poloxamer gels whose viscosity values are in the range of 19,000–36,000 mPas at 4 °C [49,50]. Therefore, the solution state with a less viscous character of IM-loaded rosin/CO-based ISGs exhibits the potential minimized painful application via injection through the needle as confirmed with subsequent injectability test.

### 2.2. Injectability and Mechanical Properties

The prepared formulations’ injectability through 21- and 24-guage needles is displayed in Table 1. The data were obtained from the area under the curve of the force/distance profile [51]. The formulations without the addition of CO were more easily injected through the 21- and 24-guage needles. Furthermore, as the rosin and CO concentrations were increased, the work required to expel the formulations through the needles also increased due to their rather high viscosity values [52]. These injectability data were consistent with our previously reported oil-incorporated ISG systems. The addition of peppermint or lime peel oil increased the force of injection of the ISG system significantly [24,25]. The oil inclusion also enhanced the injection force of the in situ forming micro-particle system more than the in situ forming gelling system [53]. Although the ISG system with CO addition required a higher force of injection than other formulations, it was easily injected through the test needle. The mechanical properties of the transformed systems after contact with agarose in terms of hardness and adhesion for the prepared ISG systems are presented in Table 1. It was found that both parameters were markedly dependent on the rosin concentration and the addition of CO. The hardness was significantly higher (*p* < 0.05) as the concentration of rosin was increased. In contrast, the addition of additives such as CO and the ISG system saw a noticeable change in hardness. The hardness declined significantly (*p* < 0.05) from 0.437 to 0.116 N, which increased the CO concentration from 2.5% (IM-RC2.5) to 10% (IM-RC10), respectively. Reduced hardness was evident due to the plasticizing effect of oil-added rosin-based ISG compared to that without incorporating oil as previously reported [45]. Therefore, the prepared CO-added rosin-based ISG system was not as hard when compared to the 55% rosin (IM-R55) ISG. Although the ISG systems loading CO were more difficult to be injected, which corresponded with their viscosity values, they were easily injected through the test needle, indicating they were easily injected at the application site. It has been reported that an injected force less of than 25 N was presented as an easy administration by the injection of dosage forms [54].

The adhesion of the rosin system was enhanced slightly after increasing the rosin concentration. Rosin is an adhesive substance used as a copolymer to improve adhesiveness [55]. In this study, the combination of rosin and 10% CO exhibited a better adhesion than an ISG system without CO. Nonetheless, 2.5 and 5% CO-included rosin-based ISGs did not show a significant difference in adhesion compared to IM-R55. The in situ forming rosin-based microparticle system using olive oil as the external phase of emulsion template showed a higher adhesion force than its rosin-based ISG system [56]. It was found that the oil promoted the adhesion of the rosin-based ISG formulation. The adhesion property of the 10% CO-added rosin ISG system was higher than the recently reported 10% lime-peel-oil-added ISG, although formulations without the oil exhibited similar values [45]. Thus, the type of oil should act differently on the adhesion properties of the ISG system. Nevertheless, the ISG system with a combination of mucoadhesive polymers also improved flow resistance and prolonged residence time at the administration site [57]. Although the recent progress was achieved in developing approaches for the mechanical reinforcement of injectable hydrogels [58], the transformation into high hardness depot promotes irritation and pain at the injection site [59]. Therefore, the lower hardness of the obtained gel from the CO-added rosin-based ISG system exhibited a potential diminishment of these side effects. The high adhesion force of CO-added ISG had a potentially longer residence time for the obtained transforming gel at the injection site [45,57].

### 2.3. Gel Formation

**Figure 2:**
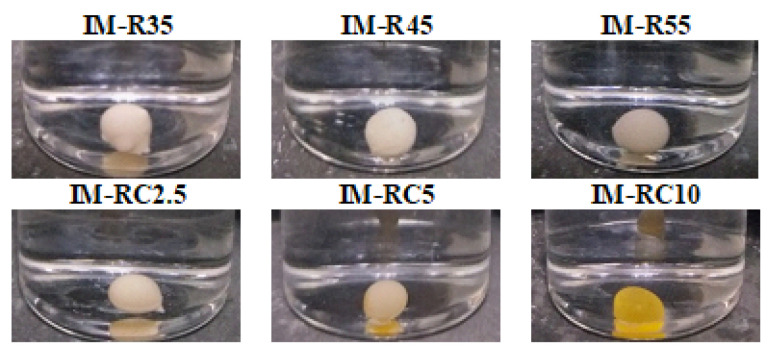
Gel formation of the IM-loaded rosin/CO-based ISG formulations after the injection of the formulations into the phosphate buffer pH 7.4.

The prepared formulations apparently formed the in situ gel after being injected into the PBS pH 7.4 buffer are shown in Figure 2. The obtained gels were spherical and white. The rosin-based ISG formulations had compact surfaces, whereas the additional CO formulations contained a soft surface and pale-yellowish color. The opaque gel was also seen for the poly- (lactic-co-glycolic acid)-based ISG after exposure to an aqueous phase due to rapid polymeric phase inversion [60,61].

**Figure 3:**
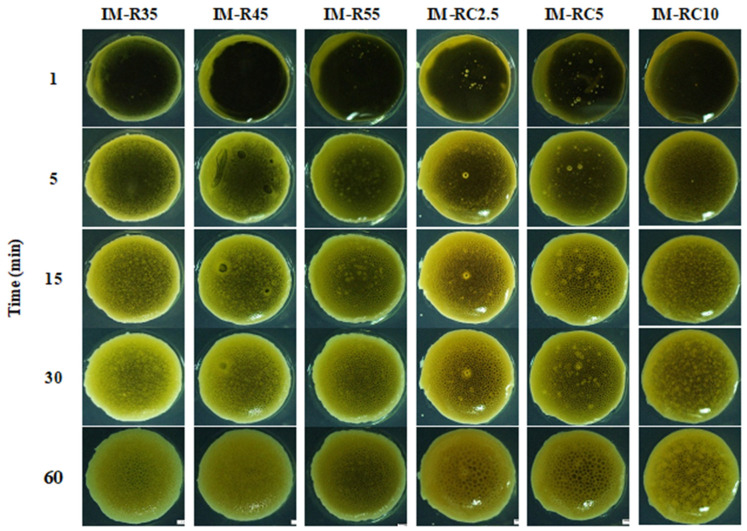
Gel formation of the IM-loaded rosin/CO-based ISG formulations after contact with agarose gel at different time intervals under using a stereomicroscope.

The cross-sectional view of the gel formation is shown in Figure 3. The rosin ISG system transformed from sol-to-gel when contact was made with an aqueous environment in the agarose well. The thickness of the gel formation was dependent on rosin and CO concentrations. All formulations presented the formation of a gel layer at 5 min after contact with an aqueous phase and showed an opaque surface layer at 30 min. The small pore formation could be observed at the surface of the CO-added formulations at 60 min. The phase inversion of the rosin ISG promoted the formation of a gel outer layer at the initial stage, and the polymer interior slowly transformed into the stable matrix over time [44]. The inclusion of CO 2.5% to 10% (IM-RC2.5-IMRC10) delayed the formation of an opaque gel surface. The rate of the aqueous penetration was also slower when the oil content was increased in the formulations. This kind of appearance was previously reported in the lime peel oil (LO)-loaded rosin-based ISG system [45]. Typically, the hydrophobic property of the oil retarded water penetration, resulting in slowing down the gel forming process [53]. Additionally, the presence of additives such as oil or mucoadhesive polymer in the ISG systems typically caused an increase in the viscosity of the prepared system and retarded gel formation [23]. This slow phase inversion could affect the gel morphology as described previously. In addition, the release of a loaded drug might be changed [61,62]. The results of gel formation were consistent with the previous viscosity results, indicating that a high viscous system owing to oil incorporation diminished the rate of phase separation of rosin into gel via solvent exchange. This slower in situ gel formation should prohibit the obstruction at needle tip during injection owing to a too rapid phase separation of the formulation [46,63].

### 2.4. Interfacial Phenomena

**Figure 4:**
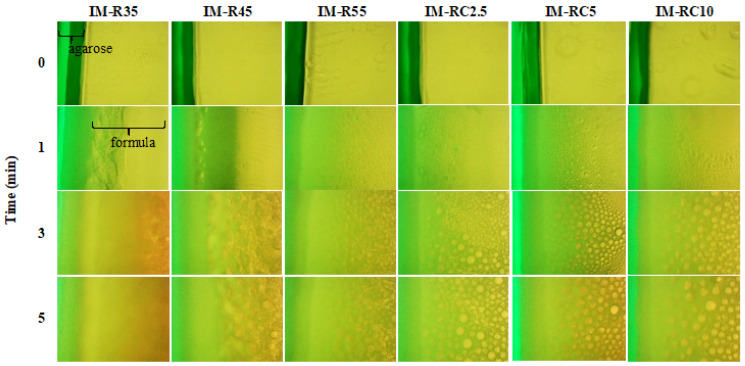
Interfacial phenomena changes of rosin in situ gel with different concentrations of CO after contact with an aqueous agarose gel phase under stereomicroscope with various time interval (40×).

Figure 4 shows the fluorescence micrographs with green and yellow regions corresponding to the agarose gel and the ISG formulation, respectively. Tracing sodium fluorescence as green color moving from the agarose was observed under a fluorescence microscope within a predetermined time [64]. The phase-separated networks of the ISG systems were attributed to the solvent exchange between these two phases. At 1 min, the movement of the green region was evident, indicating water movement into the ISG system. At 3 min, there was a miscible green/yellow region owing to the initial precipitation of the ISG components. IM-R35, presenting as a bright yellow color, did not reveal a gel structure. This low concentration of rosin ISG did not have sufficient resin to form a dense matrix after phase inversion via solvent exchange. Additionally, the antibiotic drug-loaded fatty acid (capric acid)-based ISG system did not exhibit a formation matrix owing to its liquid-like state of these saturated fatty acids. Additionally, the higher-molecular-weight saturated-fatty-acid-based ISG was unable to transform into a matrix at a low concentration after solvent exchange [64]. The IM-R45 and IM-R55 exhibited an aggregated gel structure owing to the high amount of rosin loading. When the solvent from rosin diffused outwardly, and this rosin solution exposed the inward water phase; thereafter, the transformation as small aggregates occurred simultaneously [56].

The addition of CO (IM-RC2.5 to IM-RC10) led to an assembly induced formation of small particles after contact with the aqueous phase. The particle formation appeared when more CO was incorporated into the ISG system, leading to an increased separation of the rosin gel into self-transforming small particles. Therefore, the addition of oil into the ISG system influenced phase separation into gel [15,16] and the gel formation at the initial period played an important role in understanding the microstructure. Practically, the nucleation is provoked at the interface between two phases, inducing the creation of initial solid phase [56]. Thus, these interfacial phenomena could signify the microscopic self-forming behavior of the rosin/CO gel. The profound understanding of gel formation behavior is beneficial for the modulation of drug release [64].

### 2.5. In Vitro Drug Release

#### 2.5.1. Effect of the Rosin Concentration

**Figure 5:**
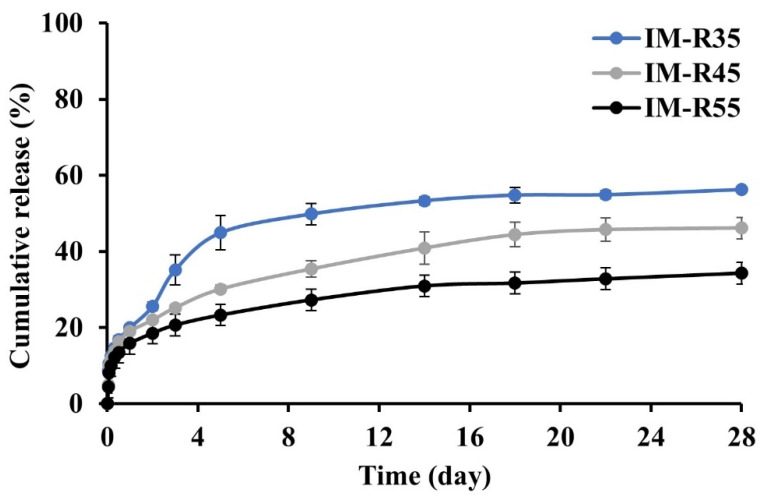
Effect of rosin concentration (35%, 45% and 55%) on IM release from IM-loaded rosin-based ISG formulations (*n* = 6).

The cumulative percentage of IM released from the rosin-based ISG formulations containing different rosin concentrations was determined, as shown in Figure 5. In the first 24 h, the rosin-based system without the additional CO formulations (IM-R35, IM-R45 and IM-R55 ISG systems) showed the IM cumulative release of 20%, 19% and 16%, respectively. Apparently, the enhanced rosin concentration in ISGs diminished IM release. The IM-R35 formulations displayed an IM release of more than 55% in the final days, whereas IM-R45 and IM-R55 were at nearly 50% and 30%, respectively. Nevertheless, all the rosin ISGs minimized the burst-IM release effect, and a more sustainable drug release could be attained up to 28 days. Typically, the drug release from the ISG system containing low concentrated polymer is faster than that of a high concentration [28,65]. Increasing the polymer concentration decreases the drug diffusion in the release medium during the solidification process by increasing the mass of the matrix as a barrier to drug diffusion [26]. Decreasing the burst drug release from the rosin–ISG systems can be explained by the rapid transformation into a gel or solid-like matrix after contact with a dissolution medium as previously described in Figure 2. Due to direct contact with the dissolution medium, the quick transformation into rosin gel was promoted and the hardening of the surrounding shell occurred. In addition, the hard surface layer efficiently diminished water diffusion and retarded drug release from the obtained gel, resulting in a minimized burst drug release and a prolonged drug release. The result is similar to the ISG system based on sucrose acetate isobutyrate-polylactic acid (SAIB-PLA), which formed a film-like structure on the spherical surface and prevented the burst release of the drug [66].

#### 2.5.2. Effect of the CO Content

**Figure 6:**
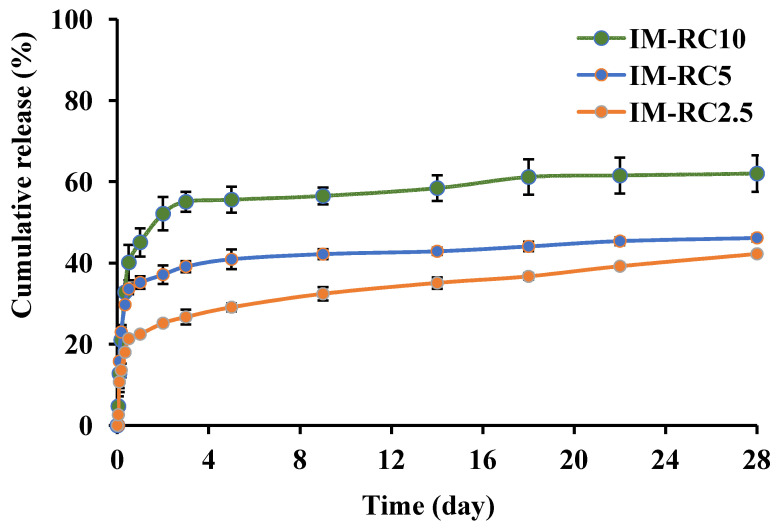
Effect of CO concentrations (2.5%, 5% and 10%) on the IM release from IM-loaded 55% *w/w* rosin/cinnamon oil-based ISG formulations (*n* = 6).

The effect of the CO content on the IM release profile is shown in Figure 6. The 55% rosin ISG formulations with CO (2.5, 5 and 10%) indicated an initial burst of the drug being released followed by the release plateau. The IM-RC2.5, IM-RC5 and IM-RC10 showed the IM release of 22%, 35% and 45% on the first day, respectively. There was a high release of IM-RC10 on day one with more than 60% of it being released by day 28. The cumulative releases of the IM-RC2.5 and IM-RC5 by day 28 were lower at 40% and 45%, respectively. The ISG systems with the addition of CO promoted a faster release of IM than without the CO-added rosin ISG systems. This result corresponded to the previous report of drugs released from the oil-loaded ISG systems. The incorporation of lime peel oil promoted the amount of total drug release and initial burst drug release than the no oil-added formulation [45]. The factors affecting the burst of drug being released include porosity, polymer molecular weight and the type of drug loading [67]. In addition, the initial burst drug release of the CO-added formulations was related to the gel structure of the rosin ISG system at the initial transformation period, which displayed a particle-like gel, as mentioned previously and presented in Figure 3. The liquid crystal microstructures of the ISG system containing glycerol monooleate and glycerol dioleate exhibited the faster drug release patterns than the aggregate gel structure comprising glycerol trioleate [68]. The more lipophilic nature of gel allowed the hydrophilic drug to diffuse on the surface of the gel during the transformation period; thereafter, it increased the drug release rate. Moreover, when hydrophilic and lipophilic additives (concentration up to 5%) were added to poly(D,L-lactic-co-glycolic acid)-based ISG systems, their drug release rate was increased [69]. Hence, this study indicates that the release of the IM-loaded rosin ISG system could be enhanced by the addition of oil such as CO.

The IM release profiles from all the ISG formulations were fitted with zero order, first order, Higuchi’s and Korsmeyer–Peppas models (Table 2). Korsmeyer–Peppas model presented the best fit for the IM release data with the highest correlation coefficient (r^2^). The release exponent (*n* value) was found to be 0.488 for the IM-RC10 formula, indicating non-Fickian diffusion, and was close to the value of 0.45 for the other formulas, indicating Fickian diffusion [70]. Thus, the release mechanism from these IM-loaded rosin-based ISG formulations was mainly controlled by diffusion. 

### 2.6. Scanning Electron Microscopy (SEM)

**Figure 7:**
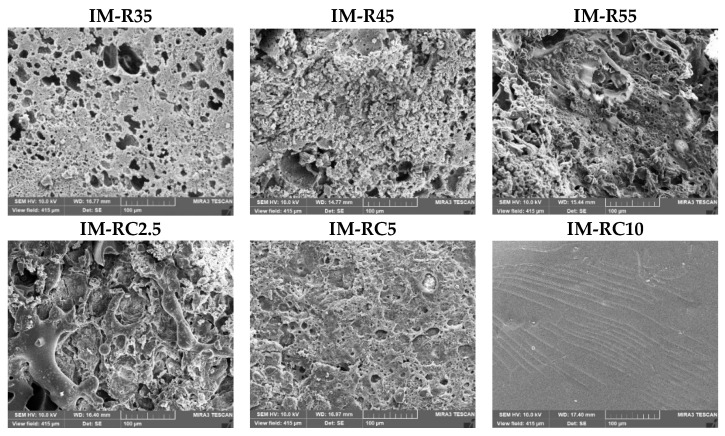
SEM images of freeze-dried IM-loaded rosin/cinnamon oil-based ISGs (500×).

The SEM images revealed the surface of the ISG formulations containing different rosin and CO concentrations (Figure 7). The concentration of the rosin had an influence on surface topography, such as an interconnected porous structure of the ISG. The IM-R35 showed a more porous structure compared to the IM-R45 and IM-R55, corresponding with more drugs being released. When the ISG solutions were injected into the aqueous solution, a rapid diffusion of DMSO moved outward into an aqueous phase via solvent exchange, resulting in the immediate formation of a porous structure with a sponge-like topography [56]. The pores were as channels for releasing the drug molecules from the inner gel mass. However, the SEM image of the CO-added ISG systems showed a noticeably different morphology. The IM-RC2.5 and IM-RC5 exhibited gel remnants with a dense and porous structure (Figure 7). Typically, the pore formation of the ISG gel was related to the solvent exchange rate. The inclusion of the CO enhanced the gels’ resistance to the diffusion of water, leading to a less porous surface [28]. The SEM image of IM-RC10 (Figure 7) demonstrated a homogenous and smooth surface without any pores. Typically, the high viscous formulations from oil incorporation absorb the small amount of water, resulting in a slow solvent diffusion with late phase transformation and attaining a smooth surface to provoke some initial burst of the drug being released [23,24]. Once the drug on the surface was depleted and the remaining drug was entrapped, drug was released much slower by diffusion through the hardened rosin matrix [46]. In addition, CO reduced the hardness of the gel as described in the mechanical investigation (Table 1). Thus, as the CO content was increased, the initial burst drug release was also increased. The similar evidence was obtained for the role of lime peel oil on the release of doxycycline hyclate from rosin-based ISG [45].

### 2.7. Cytotoxicity

#### 2.7.1. Cytotoxicity of Rosin

**Figure 8:**
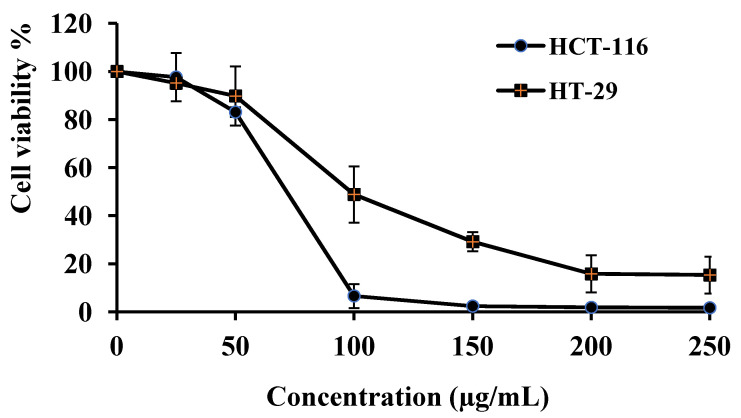
Cell viability of the HCT-116 and HT-29 cell lines after 72 h treatment with different concentrations of rosin using WST-1 assay. Results from three separate experiments: (mean ± SD).

The cytotoxicity profiles against the HCT-116 and HT-29 cell lines are presented as the percentage of cell viability after 72 h of treatment using the WST-1 assay (Figure 8). The dose-dependent cell viability was attained within the concentration range of the rosin in the treated cells. The mean inhibitory concentration of 50% (IC_50_) value was used to express the results of cytotoxic activities. Rosin exhibited the cytotoxic effect on the HCT-116 and HT-29 cell lines efficiently, with IC_50_ values of 64.60 μg/mL and 79.14 μg/mL, respectively. The results are in accordance with previous data on the cytotoxic activity of rosin against different cancer cell lines [71]. Our previous study reported IC_50_ for the cytotoxicity of IM and CO at 10.85 μg/mL and 6.51 μg/mL, respectively, for HCT-116 cells and 3.68 μg/mL and 34.67 μg/mL, respectively, for HT-29 cells [21]. These results indicate that the cytotoxic effect of the rosin was lower than that of the CO and the IM. Nevertheless, the cytotoxic activity of the rosin ISG system on colorectal cancer cells should be given further consideration.

#### 2.7.2. Cytotoxicity of ISG Formulations

**Figure 9:**
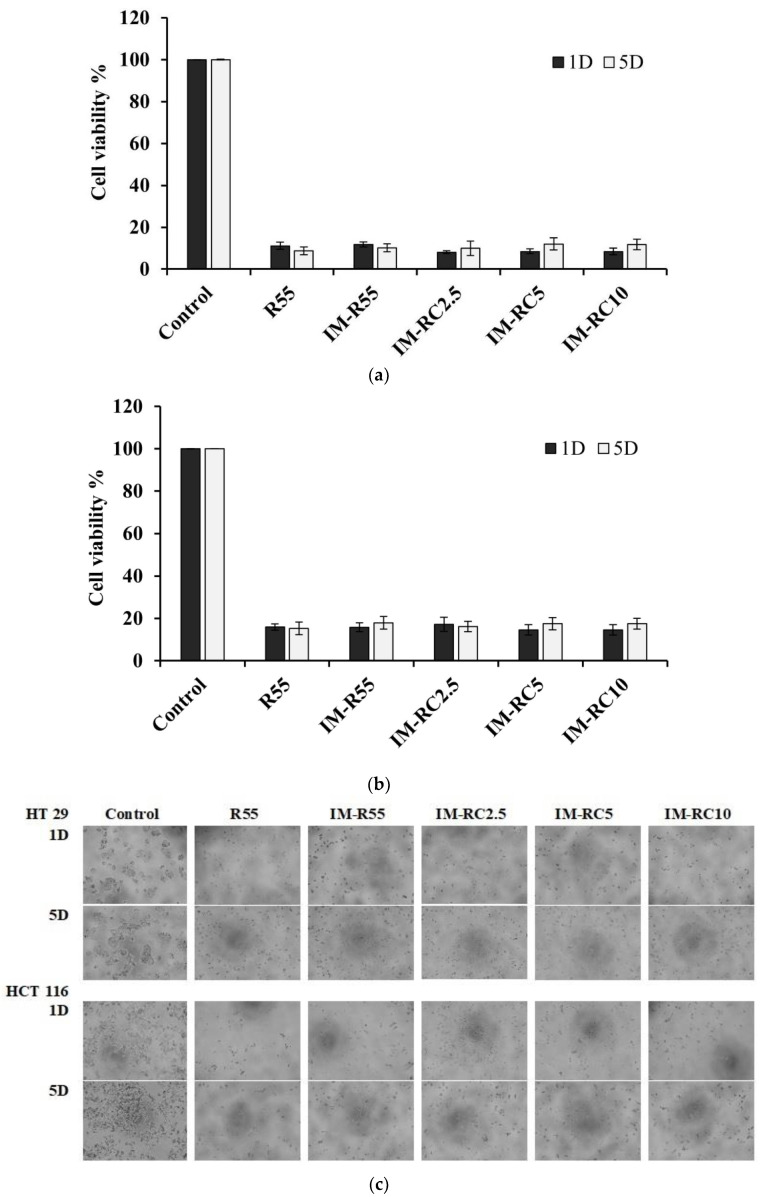
Cell viability of (**a**) HCT-116 and (**b**) HT-29 cells exposed to an extracted medium from IM-loaded rosin/CO-based ISG formulations at 1 and 5 days. (**c**) Images showing the morphology changes of the HT-29 and HCT-116 cells treated with the control and extracted medium from ISG formulations after incubation for days 1 and 5 using inverted microscope (×40 magnification).

The selected ISG formulations, both the drug-free and the IM-loaded ISG systems, were tested for cytotoxicity via the medium extraction method with slight modifications [32,35]. The drug-free rosin-based ISG formulation was used as the control group to evaluate the impact of the excipients on the cell viability. Figure 9a,b shows the cell viability of HCT-116 and HT-29 after being treated with the extracted medium of the ISG formulations with an incubation period of 1 and 5 days. There was a decrease in cell viability after exposure to the release medium from different ISG formulations for both of the tested cells. When compared to the control, the cell viability of the treated formulations was considerably lower (*p* < 0.05). Interestingly, cell viability showed no remarkable difference when comparing the drug-free (R55) and the IM-loaded formulations. The release of the IM from the extracted medium and the IM peak was confirmed from the extracted media (data not shown). As expected, the CO-loaded formulations exhibited a greater efficacy than those without CO addition; nevertheless, neither system was significant in regard to cell viability. Thus, the results suggest that the cytotoxic effect of the extracted medium simultaneously caused the IM and compounds being released from the rosin/CO ISG system. The rosin and its derivatives have been reported to have significant cytotoxic activity on human carcinoma cell lines [40]. IM is a protein tyrosine kinase inhibitor inhibiting platelet-derived growth factor receptor (PDGFR) and acting as the mechanism of action for antineoplastic activity. This drug has been commercialized and is available in oral dosage form, trademarked as Gleevec, Glivec (Novartis, Basel, Switzerland) [72]. Imatinib was reported to have an anti-proliferation effect on colon cancer cells [12,13,14]. The IC_50_ value of rosin against the HCT-116 and HT-29 cell lines was 64.60 ± 2.99 μg/mL and 79.14 ± 3.52 μg/mL, respectively. The IC_50_ value of IM against the HCT-116 and HT-29 cell lines was 10.85 ± 1.56 μg/mL and 3.68 ± 0.35 μg/mL, respectively; therefore, IM exhibited more potent action than rosin. IM showed a cytotoxic effect on HCT 116 colorectal cancer cells and the combination with selenite exhibited a synergistic action through multi-barreled molecular targeting [13]. The combination of IM and CO had a synergistic effect on colorectal cancer cell lines and improved the efficacy of IM [21]. This synergistic effect between IM and CO was stronger in HT29 than HCT116 cells. However, the applications of some oils are limited due to their low solubility; therefore, it is interesting for localized delivery near to target site with ISG. Remarkably, CO exhibited a very strong cytotoxic effect against HCT-116 cells (IC_50_ = 7.74 ± 1.29 μg/mL) and mild activity against HT-29 cells (IC_50_ = 73.95 ± 1.16 μg/mL), which had a lower IC_50_ value than IM. The result is consistent with previous reports of the IC_50_ value of CO (7.2 µg/mL) against HCT-116 [21]. The high-loading rosin was necessary for the firmness of the gel formation after solvent exchange and it exhibited the efficient inhibition of colorectal cancer cells at this rosin concentration. Thus, there was no difference in the cell viability of drug-loading or drug-free ISG in vitro cell viability determination because the release compounds easily contacted with and killed the cancel cells. Nonetheless, the in vivo accessibility of drug to the environment surrounding cancer cell is more difficult than the medium in vitro test [73,74]. Therefore, the IM-loaded formulation should exhibit its action apparently in vivo owing to IM release with its high sensitivity to cancer. From IM release profiles (Figure 6), the drug concentration was above the IC_50_ against these two colorectal cells. In addition, the different modes of antineoplastic activity from different compounds in ISG might promote or synergist the efficacy of anti-colorectal cancer.

The morphological change of the cell was employed to confirm its viability result as shown in Figure 9c. The HCT-116 and HT-29 cells, which were treated with the release medium from all the ISG formulations, showed similar characteristics, such as shrinkage and abnormal morphology, compared to those of the control group at 1 and 5 days. Additionally, there was not any significant effect on cell viability between the drugs being released at 1 and 5 days (*p* > 0.05) due to the sustained release of the IM from the ISG formulations. The results correspond with those previously reported for paclitaxel-loaded depot that showed cell viability at different incubation periods [37]. As a result, the rosin/CO-based ISG formulations could be considered for the long-term IM release for colorectal cancer chemotherapy. Nevertheless, in vivo antitumor efficacy studies are further required to investigate the prepared IM-loaded rosin ISG formulations. It should be noted that ISG formulation of 10 mg in 2 mL of culture medium were used for the medium extraction of the cell viability test. Thus, the effect of DMSO was neglectable because a small dose of DMSO was used to administration and the adverse reactions are typically related to the higher dose of DMSO [75]. Additionally, ISG system including DMSO exhibited no significant toxicity after subcutaneous administration of animal model [76]. Additionally, DMSO is recommended by American Urological Association (AUA) for the treatment of human interstitial cystitis and bladder pain syndrome, and it is conducted weekly for 6 weeks alone or combination with other medications [77]. Moreover, DMSO has been used as the solvent in pharmaceutical applications, including the injectable ISG systems for the preparation of beta-cyclodextrin [53], lauric acid [64] and poly (lactide-coglycolide) (PLGA)-based in situ forming drug delivery systems [60,75].

## 3. Conclusions

The sustained release of IM was designed using the combination of rosin and CO with the ISG system. Newtonian flow behavior was observed in all developed rosin-based ISG formulations as well as acceptable viscosity and injectability. Drug release could be modulated by varying rosin concentration and CO content. The prolongation of IM release was attained for 28 days. Under microscopic examination, the addition of the CO revealed a small particle-like appearance. Additionally, the developed IM-loaded ISG systems showed efficient cytotoxicity against the HCT-116 and HT-29 cells and the drug-free ISG was seen as well. Therefore, this study demonstrated that the designed IM-loaded rosin/CO ISG system is a promising approach for colorectal cancer drug delivery. In vivo cytotoxicity investigations should be further conducted to examine the efficacy of anti-colorectal cancer and the safety of IM-loaded rosin/CO-based ISG in animal models.

## 4. Materials and Methods

### 4.1. Materials

Rosin was purchased from Karnchanapon Co. Ltd., Nakhon Pathom, Thailand. IM was obtained from SWY Biotech Co., Ltd., Dongguan, China. CO was obtained from Thai-China Flavours and Fragrances Industry Co., LTD, Nonthaburi, Thailand. DMSO was obtained from Loba Chemie Pvt. Ltd., Mumbai, India. Acetonitrile (RCI Labscan, Bangkok, Thailand) and orthophosphoric acid (Ajax Finechem, Sydney, Australia) were used for reagent of the mobile phase. Agarose (Lot No. H7014714, Vivantis, Selangor Darul Ehsan, Malaysia) and sodium fluorescence (lot no. MKCG7851, Sigma-Aldrich, St. Louis, MO, USA) were used to analyze gel formation behavior. The human colorectal cancer cell lines (HCT-116 and HT-29) were purchased from American Type Culture Collection (ATCC), USA. Dulbecco’s Modified Eagle Medium (DMEM), fetal bovine serum, L-glutamine and penicillin/streptomycin were obtained from Gibco, Grand Island, NY, USA.

### 4.2. Preparation of ISG

For the rosin-based ISG system, IM (1%) was dissolved in DMSO. Then, various concentrations of rosin (35, 45 and 55% *w*/*w*) were added to the drug solution. The CO concentrations of 2.5, 5 and 10% *w*/*w* were added to a fixed concentration of rosin (55% *w*/*w*) preparation. The prepared solutions were mixed using a magnetic stirrer at room temperature until completely dissolved. The components of the IM-loaded ISG formulations are shown in Table 3.

### 4.3. Rheology and Viscosity Characterization

The rheological properties and viscosity of the ISG formulations were determined using a LAMY cone-plate viscometer (RM 100 CP2000 plus, LAMY RHEOLOGY, Champagne au Mont d’Or, France) equipped with a stainless spindle CP6020 at various shear rates at 25 °C. A 0.5 mL sample was used for the measurement and the experiments were conducted in triplicate.

### 4.4. Injectability and Mechanical Properties

The injectability of ISG formulations was tested using a texture analyzer in a compression mode (TA.XT Plus, Stable Micro Systems, Godalming, UK). The prepared ISG formulations were filled into a 1 mL syringe with 21-gauge and 24-gauge needles, which were clamped to a stainless-steel stand for this analysis. The upper probe of the instrument was forced downward at a constant speed (1.0-mms^−1^), and a force of 0.1 N was compressed to the syringe barrel base. The area under the resulting curve was recorded for work of injection (N.mm). These experiments were conducted in triplicate.

The mechanical properties of the ISG systems were determined using a texture analyzer (TA.XT Plus, Stable Micro Systems, Godalming, UK). The prepared 0.6 %*w*/*w* agarose was made into a well for filling formulations of 150 µL and kept for 3 days for the complete phase inversion of ISG. Then, an analytical probe of the texture analyzer was driven downwards into the obtained gel at a rate of 0.5 mm/s. This position was held for 60 s after which the probe was driven upwards at a speed of 10 mm/s. The force of the probe sample which was measured at maximum penetration was called the maximum deformation force (the hardness) and the upward movement of the probe between the surface of the sample and the probe was recorded as adhesion force [78]. The measurement was performed in triplicate.

### 4.5. Gel Formation Study

The gel formation was observed macroscopically by injection-prepared formulations through a 1 mL syringe of a 24-guage stainless needle into PBS (pH 7.4). The image indicating the transformation of the solution into the gel was taken at 5 min [45]. The formulations were investigated as to the rate of gel formation under a stereomicroscope (SZX10, Olympus Corp., Tokyo, Japan). Agarose gel (0.6% *w*/*w*) was prepared by dissolving in PBS pH 7.4 at 60 °C, and the solution was poured into Petri dishes. The 6 mm diameter wells of agarose gel were fabricated and then filled with a 150 µL sample. The gradual gel formation as a turbid/opaque ring surrounding the agarose rim occurred over time following the aqueous phase from the diffused agarose and induced a phase separation of the formula as reported previously [46]. This transformation was recorded at different time intervals (1, 5, 15, 30 and 60 min).

### 4.6. Interfacial Phenomena

Firstly, the agarose gel was prepared with a phosphate-buffered solution (pH 7.4) comprising 0.4 µg/mL sodium fluorescein to track the movement of color. The heated agarose gel (3 mL was poured onto a glass slide. To observe the interface between the aqueous phase from agarose and the ISG formulations, this agarose gel was cut to the edge and 50 µL of formulations were placed nearby. The interface was investigated under an inverted fluorescent microscope (TE-2000U, Nikon, Kawasaki, Japan) by capturing the image at different time intervals (0, 1, 3 and 5 min) [45].

### 4.7. In Vitro Drug Release Studies

The in vitro drug release study for the ISG formulations was investigated using a direct injection method. Samples of 0.05 g IM-loaded ISG formulations were injected into the 10 mL PBS (pH 7.4) and using a benchtop shaker incubator (Model NB-205, N-Biotek, Gyeonggi-do, South Korea) for orbital shaking at 37 °C with a rotational speed of 50 rpm. The formulation subsequently became gel-like upon injection. The 2 mL of release medium was sampled at specific time points and an equal volume of fresh PBS buffer was replaced to maintain the sink condition. The IM content in the release medium was analyzed (*n* = 6) using high-performance liquid chromatography (Agilent 1220 Infinity, Santa Clara, CA, USA) at 263 nm using C_8_ column (150 × 4.6 mm, 5 µm particle size, Dr. Maisch GmbH, Munich, Germany). A mobile phase consisted of acetonitrile 0.1% othophosphoric acid (17:83) and a flow rate of 1 mL/min. The IM standard (0–50 µg/mL) dissolved in the release medium was used to prepare the calibration curve for drug analysis.

The drug release mechanism was determined by fitting the obtained IM dissolution data with mathematical models, such as zero order, first order, and Higuchi’s and Korsmeyer–Peppas models. The DD-Solver software application, an add-in program for Microsoft Excel (Redmond, WA, USA) written in visual basic applications, was used to determine the drug release mechanism. The *n*-value from the Korsmeyer–Peppas equation was used to indicate the mechanism of drug release [79].

### 4.8. Scanning Electron Microscopy (SEM)

The morphology of the rosin-based ISG system was investigated with the method as previously reported [80], using SEM (TESCAN MIRA3, Tescan Orsay Holding, Brno-Kohoutovice, Czech Republic) at an accelerating voltage of 15 kV. After the drug release experiment in PBS (pH 7.4), the gel remnant was washed with 200 mL distilled water and freeze-dried. The dried samples were kept in a desiccator for 72 h and coated with gold prior to examination.

### 4.9. Cytotoxicity

Cytotoxicity of the rosin and extracted medium from the ISG formulations was investigated using the WST-1 cell viability assay with a slight modification [21]. The HCT-116 and HT-29 cells were cultured in DMEM medium containing fetal bovine serum, L-glutamine, and penicillin/streptomycin at 37 °C with a humidified 5% CO_2_ incubator (HERA Cell 240, Heraeus Deutschland GmbH & Co., Heraeus Hanau, Germany). The cell culture medium was replaced every 3 days. Briefly, the colorectal cells (0.5 × 10^3^ cells/well) were seeded in 96-well plates and incubated at 37 °C for 24 h. Following the 24 h incubation, the cell culture media were removed, and 100 μL of samples were added for 72 h. The formulation formed into gel in 5 mL of DMEM, and then the medium was collected on day 1 and day 5. Samples of six dose concentrations of rosin and extracted medium from the ISG systems were individually treated with HCT-116 and HT-29 cells. Triplicate runs were performed for each concentration sample. The cell culture medium was used as a negative control. Then, 10μL WST-1 solution was added into each well for 30 min. After incubation, the absorbance was determined at wavelength of 450 nm using a microplate reader (Victor Nivo^TM^, PerkinElmer, Hamburg, Germany). GraphPad Prism software 5.01 (San Diego, CA, USA) was used for the calculation of IC_50_ values of the cytotoxicity of rosin from three independent experiments.

### 4.10. Statistical Analysis

All data were examined using a one-way analysis of variance (ANOVA) followed by Tukey’s test. A value of *p* < 0.05 was considered statistically significant. The analysis was conducted using SPSS for Windows (version 11.5, SPSS: An IBM Company, Chicago, IL, USA).

## Figures and Tables

**Table 1:** Characterization of the injectability and mechanical properties of IM-loaded rosin/CO-based ISG formulations. (*n* = 3).

Formula	Work of Injectability (N.mm)		Hardness	Adhesion
	21-Guage	24-Guage	(*n*)	(*n*)
IM-R35	4.90 ± 0.37	13.21 ± 1.57	0.275 ± 0.03	0.028 ± 0.02
IM-R45	7.39 ± 0.50	24.91 ± 1.34	0.433 ± 0.03	0.030 ± 0.01
IM-R55	16.16 ± 0.98	84.08 ± 8.31	0.580 ± 0.02	0.040 ± 0.00
IM-RC2.5	18.71 ± 1.35	104.1 ± 5.83	0.437 ± 0.03	0.037 ± 0.01
IM-RC5	23.33 ± 3.04	125.0 ± 11.3	0.204 ± 0.03	0.042 ± 0.01
IM-RC10	27.10 ± 2.82	154.6 ± 10.0	0.116 ± 0.02	0.128 ± 0.00

**Table 2:** The regression coefficient (r^2^) value and diffusion exponent value (*n*) obtained from the IM-loaded rosin/CO-based ISG formulations.

Formula	Zero Order	First Order	Higuchi’s	Korsmeyer–Peppas		
	r^2^	r^2^	r^2^	r^2^	*n*	Release Mechanism
IM-R35	0.799	0.814	0.938	1.000	0.239	Fickian diffusion
IM-R45	0.811	0.826	0.945	1.000	0.251	Fickian diffusion
IM-R55	0.840	0.854	0.961	0.988	0.325	Fickian diffusion
IM-RC2.5	0.820	0.846	0.941	0.956	0.379	Fickian diffusion
IM-RC5	0.784	0.833	0.908	0.921	0.400	Fickian diffusion
IM-RC10	0.895	0.942	0.974	0.975	0.488	non-Fickian diffusion

**Table 3:** Composition of the IM-loaded rosin/cinnamon oil-based ISG formulations.

Formula	IM	Rosin	CO	DMSO
	(% *w*/*w*)	(% *w*/*w*)	(% *w*/*w*)	(% *w*/*w*)
IM-R35	1	35	-	64
IM-R45	1	45	-	54
IM-R55	1	55	-	44
IM-RC2.5	1	55	2.5	41.5
IM-RC5	1	55	5	39
IM-RC10	1	55	10	34

## Data Availability

The data presented in this study are available upon request from the corresponding author.
